# The Impact of Do-Not-Resuscitate Order in the Emergency Department on Respiratory Failure after ICU Admission

**DOI:** 10.3390/healthcare10030434

**Published:** 2022-02-25

**Authors:** Ting-Yu Hsu, Pei-Ming Wang, Po-Chun Chuang, Yan-Ren Lin, Yuan-Jhen Syue, Tsung-Cheng Tsai, Chao-Jui Li

**Affiliations:** 1 Department of Emergency Medicine, Kaohsiung Chang Gung Memorial Hospital, Chang Gung University College of Medicine, Kaohsiung 833, Taiwan; rs50723@gmail.com (T.-Y.H.); bogy1102@cgmh.org.tw (P.-C.C.); qoo921@cgmh.org.tw (T.-C.T.); 2 Department of Family Medicine, Kaohsiung Chang Gung Memorial Hospital, Kaohsiung 833, Taiwan; wangpeming@yahoo.com.tw; 3 Department of Emergency and Critical Care Medicine, Changhua Christian Hospital, Changhua City 500, Taiwan; h6213.lac@gmail.com; 4 School of Medicine, Kaohsiung Medical University, Kaohsiung 807, Taiwan; 5 Department of Anesthesiology, Kaohsiung Chang Gung Memorial Hospital, Chang Gung University College of Medicine, Kaohsiung 833, Taiwan; gorgeous1201@hotmail.com

**Keywords:** emergency department, do-not-resuscitate, respiratory failure, intensive care unit, medical fee

## Abstract

(1) Background: It has been hypothesized that a discrepancy exists in the understanding of a do-not-resuscitate (DNR) order among physicians. We hypothesized that a DNR order signed in the emergency department (ED) could influence the patients’ prognosis after intensive care unit (ICU) admission. (2) Methods: We included patients older than 17 years, who visited the emergency department for non-traumatic disease, who had respiratory failure, required ventilator support, and were admitted to the ICU between January 2010 and December 2016. The associations between DNR and mortality, hospital length of stay (LOS), and medical fees were analyzed. Prolonged hospital LOS was defined as hospital stay ≥75th percentile (≥26 days for the study). Patients were classified as those who did and did not sign a DNR order. A 1:4 propensity score matching was conducted for demographics, comorbidities, and etiology. (3) Results: The study enrolled a total of 1510 patients who signed a DNR and 6040 patients who did not sign a DNR. The 30-day mortality rates were 47.4% and 28.0% among patients who did and did not sign a DNR, respectively. A DNR order was associated with mortality after adjusting for confounding factors (hazard ratio, 1.9; confidence interval, 1.70–2.03). It was also a risk factor for prolonged hospital LOS in survivors (odds ratio, 1.2; confidence interval, 1.02–1.44). Survivors who signed a DNR order were charged higher medical fees than those who did not sign a DNR (217,159 vs. 245,795 New Taiwan Dollars, *p* < 0.001). (4) Conclusions: Signing a DNR order in the ED increased the ICU mortality rate among patients who had respiratory failure and needed ventilator support. It increased the risk of prolonged hospital LOS among survivors. Finally, signing a DNR order was associated with high medical fees among survivors.

## 1. Introduction

A do-not-resuscitate (DNR) order was first published in the medical literature in 1976 [1]. It is a concept derived from palliative care and literally means “on the occasion of cardiac arrest, do not perform cardiopulmonary resuscitation (CPR) and conduct other forms of life-sustaining treatments’’ [2,3]. It is an order written on a legal form either within or outside the hospital setting to withhold cardiopulmonary resuscitation or advanced cardiac life support.

Withholding or withdrawing a patient’s treatment is also influenced by a DNR order [4,5]. Practically, it broadly ranges from do-not-CPR to do-not-treat patients aggressively if the disease progresses; therefore, the patient’s prognosis is influenced by the physician’s understanding of the order. It can be hypothesized that a discrepancy may arise in the understanding of a DNR order by physicians. While treating patients who have signed a DNR, whether to withhold intensive treatment or only CPR during the development of a cardiac arrest might be a dilemma for physicians [6,7]. Previous studies reported that the mortality rate was higher in patients who signed a DNR order in the intensive care unit (ICU) [4,8]. Studies have also shown that a DNR order was associated with high mortality in patients with heart failure and sepsis [9,10,11]. Hence, whether a DNR should be determined very early, while patients are still in the emergency department (ED), remains controversial.

People with acute respiratory failure usually require immediate airway management and ventilator support in the ED. Without immediate treatment, the result is life-threatening, and sometimes fatal [12]. The decision to adhere to a DNR order can be determined before or after airway management. We believe that some patients might experience respiratory failure and sign a DNR order, following which intubation and ventilator support are withheld, and eventually the patient may die soon after. Other patients on ventilator support could survive and be transferred to the ICU, and then be influenced by the DNR order. Some emergency physicians (EPs) might hesitate to disposition respiratory patients with a DNR order. Concerning the limitations of medical resources, such as ICU beds, especially during the coronavirus disease (COVID-19) pandemic, it remains debatable whether a patient receiving palliative treatment should be admitted to the ICU for ventilator care alone [13]. A previous study stated that admitting patients who signed a DNR order to the ICU before admission was potentially a misallocation of limited resources to patients who may neither need nor want intensive care [14]. Limited studies have discussed the influence of DNR orders on these patients. In this study, we aimed to investigate the prognosis of such patients. We hypothesized that a DNR order signed in the ED could influence the patients’ prognosis after ICU admission.

## 2. Materials and Methods

### 2.1. Study Setting

The data were obtained from the largest healthcare system in Taiwan, Chang Gung Memorial Hospital. The Chang Gung Research Database (CGRD) is an original, multi-institutional medical record-based research database [15]. In this study, the data from Keelung, Linkou, Chiayi, and Kaohsiung branches, located from northern to southern Taiwan, were analyzed. All patient records and information were anonymized and deidentified before the analysis.

### 2.2. Study Participants

In this study, we included all patients aged 17 years and older, who visited the ED for non-traumatic disease, experienced respiratory failure on ventilator support, and were then admitted to the ICU between January 2010 and December 2016. Patients with out-of-hospital cardiac arrest (OHCA) were excluded. Patients who had signed a DNR order in the ED were classified as the DNR group, and the others were placed into the non-DNR group.

### 2.3. Measurements

Data on patient demographics, comorbidities, and medical records were extracted from the CGRD. Patients were divided into five diagnostic groups based on the cause of respiratory failure, including nervous, circulatory, respiratory, digestive, and other metabolic conditions. Patients were grouped according to the diagnostic codes from the International Classification of Diseases, Ninth and Tenth Revision, Clinical Modification. A DNR order in the ED was written by the patient’s primary care visiting staff and confirmed by the other visiting staff after realizing the willing of the patient or family. A DNR order specified that cardiopulmonary resuscitation should not be performed in case of a cardiac arrest, but patients could still receive other treatments, such as inotropic agent or renal treatment. In-hospital mortality was defined as death occurring in the hospital within 30 days, which was the primary outcome of the study. The secondary outcomes were length of hospital stay (LOHS) and medical fee.

### 2.4. Data Analysis

Continuous variables, such as age, are presented as mean ± standard deviation. The hospital LOS and medical fee are presented as median. Prolonged hospital LOS was defined as the proportion of the study participants with hospital stay greater than the 75th percentile (≥26 days for the study) [16]. Categorical data are presented as numbers and percentages. The student’s t-test, the Mann–Whitney U test, and the chi-square test were used for data analysis. A logistic regression analysis was conducted to analyze the correlation between patients’ demographic characteristics and the DNR order.

### 2.5. Propensity Score Matching (PSM)

The propensity score was calculated using logistic regression. Variables, including age, sex, comorbidities (myocardial infarction, heart failure, peripheral arterial disease, cerebrovascular accident, chronic obstructive pulmonary disease, peptic ulcer disease, liver cirrhosis, diabetes mellitus, chronic kidney disease, and malignancy), and main diagnoses, were used to estimate the probability of signing a DNR order in the ED. The main diagnoses, which were divided into nervous, circulatory, respiratory, digestive, and other metabolic conditions, were considered to cause respiratory failure in a single episode. PSM was performed using NCSS version 12.0.4 (NCSS statistical software, LLC, Kaysville, UT, USA). The greedy method was used to create a 1:4 matched study group with a 0.25 SD width. The caliper half-width was 0.17579, and the average matched Mahalanobis distance was 0.00014.

To determine the predictors of mortality in patients who signed a DNR order, survival analysis with Cox regression was performed. The effects were estimated using hazard ratios (HRs) and corresponding 95% confidence intervals (CIs). To identify predictor variables for prolonged hospital length of stay, multivariate logistic regression was performed. The effects were estimated using odds ratios (ORs) and corresponding 95% CIs. Statistical significance was set at *p* < 0.05. In addition to PSM, IBM Statistical Package for the Social Sciences for Windows version 22.0 (released 2013, IBM Corp., Armonk, NY, USA) was used for statistical analyses. A similar statistical method of PSM and Cox regression was also conducted in a recently published article [17].

## 3. Results

**Figure A1:**
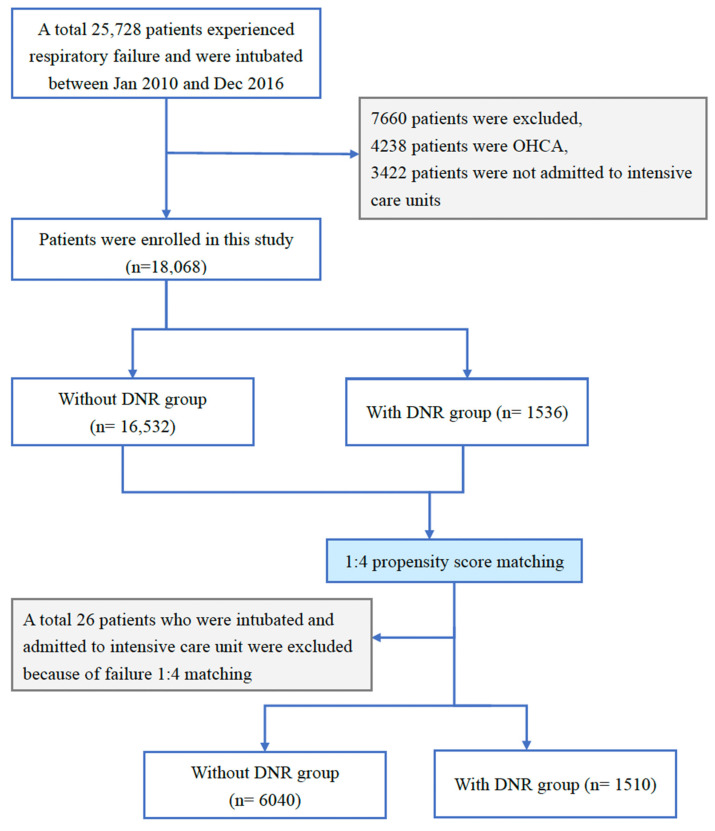
Flowchart of enrollment and status of patients. The 1:4 propensity score matched by age, sex, myocardial infarction, heart failure, cerebrovascular accident, chronic obstructive pulmonary disease, liver cirrhosis, chronic kidney disease, malignancy, and main diagnosis. Abbreviations: OHCA, out-of-hospital cardiac arrest; DNR, do-not-resuscitate.

A total of 25,728 patients with respiratory failure had visited the ED during the study period. After excluding patients with out-of-hospital cardiac arrest and those who were not admitted to the ICU, 18,068 patients were enrolled. Among them, 1536 patients signed a DNR (Figure A1, see Appendix A). The demographic characteristics of patients who did and did not sign a DNR are presented in the Table A1 (see Appendix B). Table A2 (see Appendix B) shows the relationship between patients’ demographic characteristics and a DNR order. Patients with increasing age, cerebrovascular disease, chronic kidney disease, and malignancy were more likely to sign a DNR order, while patients with chronic ischemic heart disease and chronic obstructive pulmonary disease were less likely to do so. After a 1:4 PSM, the demographics and distribution of comorbidities between 1510 patients who signed a DNR order and 6040 patients who did not sign a DNR order were similar (Table 1).

**Figure 1:**
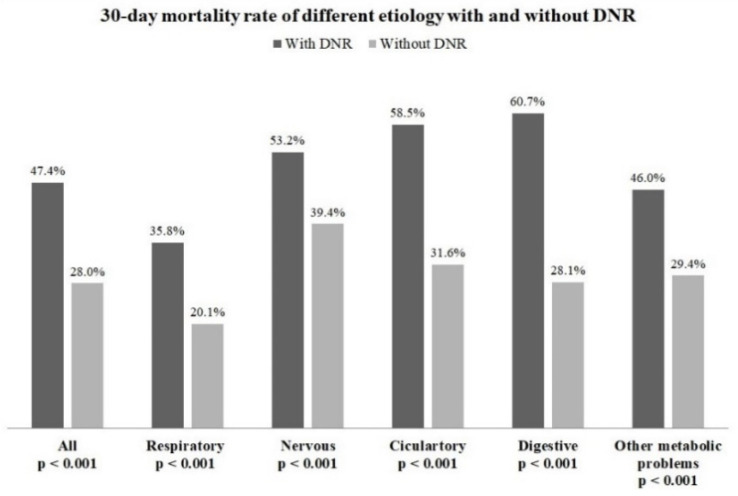
The 30-day mortality rate of different etiology with and without DNR after 1:4 propensity score matching.

The 30-day mortality rates were 47.4% among patients who signed a DNR order and 28.0% among those who did not. Figure 1 shows the 30-day mortality rates in patients who did and did not sign a DNR order in the five main groups with diseases that caused respiratory failure. After PSM, the 30-day mortality rate was higher in patients who signed a DNR order in the five groups than those who did not. A Cox regression analysis was conducted to determine the association between a DNR order and mortality. A DNR order was associated with mortality after adjusting for age, sex, comorbidities, and causes of admission (adjusted HR, 1.9; 95% CI, 1.70–2.03). After stratifying the five causes of admission, a DNR order was found to influence the outcomes in all the patients (Table 2). Further survival analysis was conducted in patients who signed a DNR order. Table 3 shows the predictors of mortality in patients who signed a DNR order. Those with cerebrovascular disease, liver cirrhosis, chronic kidney disease, and malignancy had a relatively high risk of mortality. Patients admitted due to the respiratory problems had a relatively low risk of mortality.

**Figure 2:**
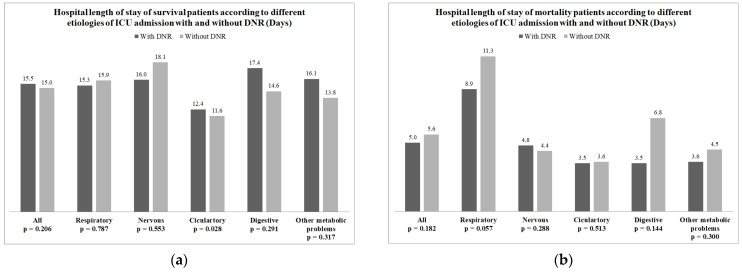
(**a**) The median days of hospital length of stay of all patients who survived and those with different etiology for intensive care unit (ICU) admission after 1:4 propensity score matching; (**b**) the median length of hospital stays of all patients who died and those with different etiology for ICU admission after 1:4 propensity score matching.

The overall median hospital LOS of patients who signed a DNR order was shorter than that of patients who did not sign a DNR order (8.3 days vs. 12.1 days, *p* < 0.001). After stratifying survival and patients who died, there was no difference in the median hospital LOS between patients who did and did not sign a DNR among those who survived (15.5 days vs. 15.0 days, *p* = 0.206) and died (5.0 days vs. 5.6 days, *p* = 0.182). The median hospital LOS for different causes of ICU admission for survivors and patients who died are presented in Figure 2a,b, respectively. Among the survivors who were admitted to the ICU due to circulatory conditions, the median hospital LOS was longer in patients who signed a DNR order than in those who did not (12.4 days vs. 11.6 days, *p* = 0.028). Table 4 shows the predictors of prolonged hospital length of stay by multivariate logistic regression. Chronic kidney disease, malignancy, do-not-resuscitation order and diseases of the nervous system were associated with prolonged hospital length of stay.

**Figure 3:**
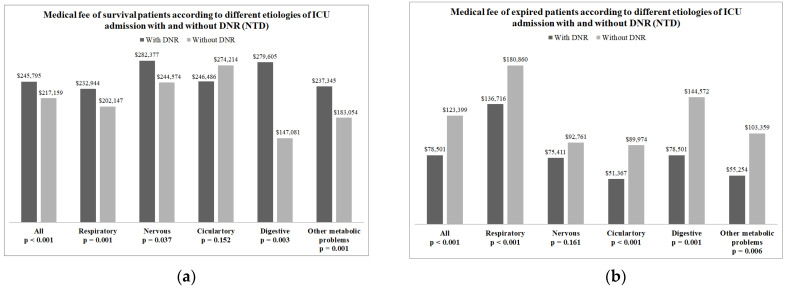
(**a**) The median medical fee (NTD) of all patients who survived and those with different etiology for ICU admission after 1:4 propensity score matching; (**b**) the median medical fee (NTD) of all patients who died and those with different etiology for ICU admission after 1:4 propensity score matching. NTD: New Taiwan Dollar.

The overall medical fee of patients who signed a DNR order was lower than that of patients who did not sign a DNR order (173,488 vs. 192,597 New Taiwan Dollars, *p* < 0.001). After stratifying survivors and expired patients, patients who signed a DNR order had higher medical fees than those who did not sign a DNR order, except patients with a disease of the circulatory system (Figure 3a). In contrast, among the patients who died, the medical fee was higher in those who did not sign a DNR order than those who signed a DNR order (Figure 3b).

## 4. Discussion

Recently, some studies have reported the impact of DNR orders on the prognosis of patients. Since the determination of DNR is often related to the severity and progression of the disease [18], comparing the prognosis between patients who did and did not sign a DNR might be biased due to their clinical condition. To control these confounding factors, we conducted a PSM analysis. This helped us analyze the influence of a DNR order on similar demographics in the two study groups. After PSM, we found that the mortality rate of patients who signed a DNR order was 19.4% higher than those who did not sign a DNR order. The difference still existed after stratifying based on various diagnoses. According to a previous study, the 28-day mortality rate was higher among ICU patients who signed a DNR order on the first day of ICU admission and survived for more than 48 h as compared to those who did not sign a DNR order (33.9% vs. 18.4%) [8]. The mortality rates in our study were 47.4% vs. 28.0%. This difference in the mortality rate may be due to the fact that Fuchs et al. excluded patients who died within the first 48 h of ICU admission [8]; however, in our study, all deaths that occurred in the ICU were included. As compared to previous studies, since there was an obvious difference in the mortality rate between patients who did and did not sign a DNR order, we concluded that a DNR order signed in the ED could influence patient’s mortality after ICU admission.

A previous study declared that certain categories of patients might be keen to sign a DNR. Fuchs et al. also reported that women and cancer patients are particularly likely to sign a DNR order [8]. They suggested that the physician should adjust the treatment according to personal perceptions of the patient characteristics. They emphasized that the association between a DNR order and mortality was 50% higher in women than in men. However, in our study, although patients who signed a DNR order were at risk for mortality if they were diagnosed with cerebrovascular disease, chronic kidney disease, liver cirrhosis, or malignancy; sex was not associated with mortality. In this study, we hypothesized that physicians might alter treatment according to their own perception based on the patients’ clinical condition, but not their gender.

Previous studies have reported that patients who sign a DNR order early upon ICU admission also have shorter LOHS than those who were later determined to sign a DNR order [4]. It was not surprising because the mortality rate of DNR patients was higher than the non-DNR patients. To control this confounding factor, in this study, we stratified the analysis into survivors and expired patients. We found that among survivors who were admitted to ICU for circulatory disease, those with a DNR order had relatively long hospital LOS than those without a DNR order. Further regression analysis showed that a DNR order was a risk factor for prolonged hospital LOS. A previous study suggested that patients with decompensated heart failure who signed a DNR order were less likely to receive pharmacologic and non-pharmacologic interventions compared to non-DNR patients [19]. We assumed that withdrawing or withholding treatment might cause prolonged hospital stay among survivors who signed a DNR order. Moreover, in our study, we also found that among ICU survivors, those who signed a DNR order paid higher medical fees than those who did not sign a DNR order. This might imply that while some intervention treatments were withheld, physicians might still let the routine treatments continue, causing an increase in the medical fee. Beach and Morrison found that physicians initiate fewer interventions in patients who sign a DNR order [6]. For instance, if a patient has gastrointestinal bleeding, but endoscopy had to be withheld due to a DNR order, this would cause prolonged medication treatment, such as proton pump inhibitor injection, increased blood transfusions, and a longer period of ICU stay. All these factors could result in an increase in the medical fees. However, due to the limitations of this retrospective study, we do not have data to support this explanation. Further studies are required to clarify and validate our findings. To avoid the adverse effect of DNR in ED is important. Recently, a Belgian/French societies’ consensus conference has some suggestion on management of cancer patients in the ICU [20]. According to these guidelines, we believed that the discrepancy between physicians’ treatment on terminal patients could be diminished. It might also reduce the prolonged hospital LOS and medical fees in the DNR patients.

This study had some limitations. First, this study documented DNR orders in the ED. We believe that some patients may choose to sign or withdraw a DNR after admission. This might influence the prognosis of the patient. Second, the four study settings belonged to the same medical system, limiting the implications of the conclusions. Finally, considering the retrospective study design, there might be some confounding factors that were not measured in the analysis, which may have influenced the study outcomes.

## 5. Conclusions

Signing a DNR order in the ED increased the rate of ICU mortality in those who had respiratory failure and required ventilator support. It increased the risk of prolonged hospital LOS in survivors. Finally, a DNR order also increased the medical fees for survivors.

## Figures and Tables

**Table 1:** Clinical characteristics of patients after 1:4 propensity score matching.

	With DNR n = 1510	Without DNR n = 6040	*p*-Value
Age	71.53 ± 14.6	71.71 ± 14.10	0.660
Male sex	930(61.6)	3699(61.2)	0.804
Chronic ischemia heart disease	168(11.1)	626(10.4)	0.388
Cerebrovascular disease	223(14.8)	908(15)	0.796
Chronic obstructive pulmonary disease	247(16.4)	970(16.1)	0.778
Liver cirrhosis	143(9.5)	551(9.1)	0.676
Chronic kidney disease	337(22.3)	1285(21.3)	0.377
Malignancy	354(23.4)	1381(22.9)	0.632
Main diagnosis			0.834
Disease of the respiratory system	581(38.5)	2411(39.9)	
Disease of the nervous system	293(19.4)	1136(18.8)	
Disease of the circulatory system	340(22.5)	1353(22.4)	
Disease of the digestive system	107(7.1)	430(7.1)	
Other metabolic problems	189(12.5)	710(11.8)	

Data were presented as mean ± standard deviation and number (percentage). Abbreviations: DNR, do-not-resuscitate.

**Table 2:** The association between DNR order and mortality adjusted for age, sex and comorbidities by Cox regression in different diagnosis groups.

Diagnosis	aHR	95% CI of HR
All *	1.9	1.70–2.03
Disease of the respiratory system	2.0	1.71–2.36
Disease of the nervous system	1.4	1.19–1.72
Disease of the circulatory system	2.0	1.68–2.36
Disease of the digestive system	2.7	2.00–3.69
Other metabolic problems	1.7	1.30–2.16

* Adjusting for five diagnostic groups. aHR: adjust hazard ratio; CI, confidence interval.

**Table 3:** Predictors of mortality in patients with DNR order.

Variable	HR	95% CI of HR
Age	1.0	0.99–1.00
Male sex	1.0	0.84–1.14
Chronic ischemia heart disease	1.2	0.96–1.60
Cerebrovascular disease	2.7	2.11–3.41
Chronic obstructive pulmonary disease	0.8	0.62–1.02
Liver cirrhosis	1.7	1.29–2.14
Chronic kidney disease	1.3	1.12–1.59
Malignancy	1.4	1.16–1.65
Main diagnosis		
Disease of the respiratory system	1.0	
Disease of the nervous system	1.5	1.22–1.88
Disease of the circulatory system	1.3	0.10–1.65
Disease of the digestive system	1.4	1.03–1.95
Other metabolic problems	1.3	1.01–1.69

HR: hazard ratio; CI, confidence interval.

**Table 4:** Predicotors of prolonged hospital length of stay in survival patients.

Variable	OR	95% CI of HR
Age	1	0.999–1.009
Male sex	1.1	0.92–1.21
Chronic ischemia heart disease	0.7	0.59–0.95
Cerebrovascular disease	0.9	0.72–1.17
Chronic obstructive pulmonary disease	0.8	0.64–0.92
Liver cirrhosis	1	0.77–1.33
Chronic kidney disease	1.5	1.25–1.72
Malignancy	1.7	1.43–1.98
Do-not-resuscitate	1.2	1.02–1.44
Main diagnosis		
Disease of the respiratory system	1	
Disease of the nervous system	1.4	1.15–1.66
Disease of the circulatory system	0.9	0.69–1.07
Disease of the digestive system	0.8	0.59–1.08
Other metabolic problems	0.8	0.67–1.04

## Data Availability

Data were obtained from the Chang Gung Research Database and are available from corresponding author with the permission of Chang Gung Medical Foundation.

## Appendix B

**Table A1:** Clinical characteristics of patients.

	With DNR n = 1536	Without DNR n = 16532	*p*-Value
Age	71.71 ± 14.6	66.45 ± 15.7	< 0.001
Male sex	948(61.7)	10,554(63.8)	0.098
Chronic ischemia heart disease	168(10.9)	2314(14.2)	< 0.001
Cerebrovascular disease	223(14.5)	2487(15)	0.581
Chronic obstructive pulmonary disease	247(16.1)	3115(18.8)	0.008
Liver cirrhosis	147(9.6)	1756(10.6)	0.199
Chronic kidney disease	339(22.1)	3241(19.6)	0.020
Malignancy	380(24.7)	2220(13.4)	< 0.001
Main diagnosis			< 0.001
Disease of the respiratory system	589(38.3)	6078(36.8)	
Disease of the nervous system	310(20.2))	1980(12.0)	
Disease of the circulatory system	340(22.1)	4536(27.4)	
Disease of the digestive system	107(7.0)	1697(10.3)	
Other metabolic problems	190(12.4)	2241(13.6)	

Data were presented as mean ± standard deviation and number (percentage); Abbreviations: DNR, do-not-resuscitate.

**Table A2:** Logistic regression analysis for the predictors of do-not-resuscitate.

Variable	OR	95% CI of OR
Age	1.0	1.02–1.03
Male sex	1.0	0.87–1.09
Chronic ischemia heart disease	0.7	0.63–0.88
Cerebrovascular disease	1.2	1.00–1.37
Chronic obstructive pulmonary disease	0.8	0.65–0.87
Liver cirrhosis	1.1	0.87–1.27
Chronic kidney disease	1.2	1.06–1.38
Malignancy	2.3	2.03–2.63

Abbreviations: OR, odds ratio; CI, confidence interval.

## References

[1] Burns J.P., Truog R.D. (2016). The DNR Order after 40 Years. N. Engl. J. Med..

[2] Walsh E.C., Brovman E.Y., Bader A.M., Urman R.D. (2017). Do-Not-Resuscitate Status Is Associated With Increased Mortality But Not Morbidity. Anesth. Analg..

[3] Smith C.B., Bunch O’Neill L. (2008). Do not resuscitate does not mean do not treat: How palliative care and other modalities can help facilitate communication about goals of care in advanced illness. Mt. Sinai J. Med..

[4] Baek M.S., Koh Y., Hong S.-B., Lim C.-M., Huh J.W. (2016). Effect of Timing of Do-Not-Resuscitate Orders on the Clinical Outcome of Critically Ill Patients. Korean J. Crit. Care Med..

[5] Patel K., Sinvani L., Patel V., Kozikowski A., Smilios C., Akerman M., Kiszko K., Maiti S., Hajizadeh N., Wolf-Klein G. (2018). Do-Not-Resuscitate Orders in Older Adults During Hospitalization: A Propensity Score-Matched Analysis. J. Am. Geriatr. Soc..

[6] Beach M.C., Morrison R.S. (2002). The effect of do-not-resuscitate orders on physician decision-making. J. Am. Geriatr. Soc..

[7] Bedell S.E., Pelle D., Maher P.L., Cleary P.D. (1986). Do-not-resuscitate orders for critically ill patients in the hospital. How are they used and what is their impact?. JAMA.

[8] Fuchs L., Anstey M., Feng M., Toledano R., Kogan S., Howell M.D., Clardy P., Celi L., Talmor D., Novack V. (2017). Quantifying the Mortality Impact of Do-Not-Resuscitate Orders in the ICU. Crit. Care Med..

[9] Huang C.T., Chuang Y.C., Tsai Y.J., Ko W.J., Yu C.J. (2016). High Mortality in Severe Sepsis and Septic Shock Patients with Do-Not-Resuscitate Orders in East Asia. PLoS ONE.

[10] Hiraoka E., Arai J., Kojima S., Norisue Y., Suzuki T., Homma Y., Takahashi O., Obunai K., Watanabe H. (2020). Early DNR Order and Long-Term Prognosis Among Patients Hospitalized for Acute Heart Failure: Single-Center Cohort Study in Japan. Int. J. Gen. Med..

[11] Chang Y.C., Fang Y.T., Chen H.C., Lin C.Y., Chang Y.P., Chen Y.M., Huang C.H., Huang K.T., Chang H.C., Su M.C. (2019). Effect of do-not-resuscitate orders on patients with sepsis in the medical intensive care unit: A retrospective, observational and propensity score-matched study in a tertiary referral hospital in Taiwan. BMJ Open.

[12] Overbeck M.C. (2016). Airway Management of Respiratory Failure. Emerg. Med. Clin. N. Am..

[13] Cardenas H.C., Carson R.T., Hanemann M., Louviere J.J., Whittington D. (2022). Who should get the scarce ICU bed? The US public’s view on triage in the time of COVID-19. Emerg. Med. J..

[14] Saha D., Moreno C., Csete M., Perez E.K., Cubeddu L., Farcy D., Henry S., Glazer Z., Moreno-Walton L.A., Goldszer R.C. (2016). Outcomes of Patients Who Have Do Not Resuscitate Status prior to Being Admitted to an Intensive Care Unit. Scientifica.

[15] Shao S.C., Chan Y.Y., Kao Yang Y.H., Lin S.J., Hung M.J., Chien R.N., Lai C.C., Lai E.C. (2019). The Chang Gung Research Database-A multi-institutional electronic medical records database for real-world epidemiological studies in Taiwan. Pharmacoepidemiol. Drug Saf..

[16] Krell R.W., Girotti M.E., Dimick J.B. (2014). Extended length of stay after surgery: Complications, inefficient practice, or sick patients?. JAMA Surg..

[17] Li C.J., Law Y.Y., Lin Y.R., Chen C.C., Lin X.H., Chuang P.C. (2021). Impact of Using a Non-Rebreathing Mask in Patients With Respiratory Failure. Am. J. Med. Sci..

[18] Wenger N.S., Pearson M.L., Desmond K.A., Brook R.H., Kahn K.L. (1995). Outcomes of patients with do-not-resuscitate orders. Toward an understanding of what do-not-resuscitate orders mean and how they affect patients. Arch. Intern. Med..

[19] Chen J.L., Sosnov J., Lessard D., Goldberg R.J. (2008). Impact of do-not-resuscitation orders on quality of care performance measures in patients hospitalized with acute heart failure. Am. Heart J..

[20] Meert A.P., Wittnebel S., Holbrechts S., Toffart A.C., Lafitte J.J., Piagnerelli M., Lemaitre F., Peyrony O., Calvel L., Lemaitre J. (2021). Critically ill cancer patient’s resuscitation: A Belgian/French societies’ consensus conference. Intensive Care Med..

